# Comparing analysis methods in assessing dynamic dual bolus cardiac magnetic resonance perfusion flow

**DOI:** 10.1186/1532-429X-15-S1-W38

**Published:** 2013-01-30

**Authors:** BM Muller-Bierl, K Tanaka, Y Fierens, N Buls, T van Cauteren, I Willekens, R Luypaert, J De Mey

**Affiliations:** 1 Radiology, Flemish University Hospital Brussels, Brussels, Belgium; 2 Radiology, Flemish University Hospital Brussels, Brussels, Belgium

## Background

We compare eight reported methods (1-8) for the analysis of cardiac perfusion flow in 3 Tesla MRI on a porcine model. Therefore, an anaesthetized healthy minipig was repeatedly scanned (x5) with a 14 day interval with Turbo FLASH at 3T. The data obtained from the images consists of the temporal course of the arterial input function (AIF) and of the tissue response function (TRF). We compared 8 analysis methods by statistical evaluation of determined perfusion flow. The analysis methods investigated were Fermi function (1), Model-free Deconvolution (2), Modified Tofts (3), Exchange (4), Uptake (5), Tofts (6), Patlak (7), and Maximum Slope (8).

**Figure 1:**
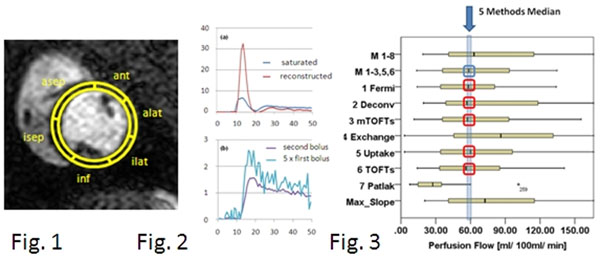
Mid-ventricular short axis view of the minipig heart with contrast agent inflow. The TurboFLASH sequence was used for imaging. The 6 standardized ROIs from the nuclear medicine 17 segment heart model are shown (cf. text). 2 : Saturated and reconstructed arterial input function (AIF (a)) and tissue response function (TRF (b)). The TRF time series owes much more noise than her AIF counterpart, due to movement of the septum not being compensated for by cardiac gating. 3 : Boxplots of perfusion flow assessed with the eight analysis methods compared to the 8 methods arithmetic mean, respectively the 5 methods arithmetic mean.

## Methods

We compared the analysis methods by correlation analysis, by comparing the medians of the measured flow values, by regression analysis, by Bland-Altman analysis, and by related samples Wilcoxon rank test. We also investigated numerically the throughput of noise in the TRF on the analysis methods 1-4.

## Results

Five methods (Fermi, Deconvolution, the Tofts methods, and Uptake) were found to be suitable for normal perfusion flow evaluation. Repeatability of the results - independent on the analysis methods - was bad, which is due to the movement of the septum. For measuring stress perfusion, numerical modeling shows that using the Fermi and modified Tofts methods results in large bias errors in the presence of noise in the TRF.

## Conclusions

As long as there is noise present in the TRF due to the moving septum, the evaluation of perfusion flow is not possible. We expect that measurement of cardiac perfusion flow will be possible using the Turbo FLASH sequence and the theoretical frameworks for perfusion flow analysis using the dual bolus method. Based on our investigation so far, we claim that Fermi, Model-Free Deconvolution, Uptake and the Tofts methods are all suitable for normal perfusion flow evaluation. Evaluation of the TRF in heart has to use spatial registration and correction: To establish a reliable procedure to assess the perfusion value based on the dual bolus data, motion correction of the septum by spatial image reconstruction is mandatory and should be the next logical step.

## Funding

The work was payed by the Department of Radiology of the Flemish University Hospital Brussels.

